# Intradiscal Injection of Minimally Manipulated Adipose Tissue for Disc-Driven Cervical Myelopathy: A Case Report With Three-Year Follow-Up

**DOI:** 10.7759/cureus.107314

**Published:** 2026-04-18

**Authors:** Yonghyun Yoon, Jihyo Hwang, Jaeyoung Lee, Jaewoo Lim, Teinny Suryadi, Anwar Suhaimi, Jeimylo C de Castro, Jonghyeok Lee, Seungbeom Kim, King Hei Stanley Lam

**Affiliations:** 1 Orthopedics, International Academy of Musculoskeletal Medicine, Hongkong, HKG; 2 Orthopedics, International Academy of Regenerative Medicine, Incheon, KOR; 3 Orthopedics, Musculoskeletal Ultrasound (MSKUS), San Diego, USA; 4 Orthopedic Surgery, Hallym University Kangnam Sacred Heart Hospital, Seoul, KOR; 5 Orthopedic Surgery, Incheon Terminal Orthopedic Surgery Clinic, Incheon, KOR; 6 Orthopedics, Incheon Terminal Orthopedic Surgery Clinic, Incheon, KOR; 7 Physical Medicine and Rehabilitation, Medistra Hospital, Central Jakarta, IDN; 8 Physical Medicine and Rehabilitation, Synergy Clinic, Jakarta, IDN; 9 Physical Medicine and Rehabilitation, Hermina Hospital Podomoro, Jakarta, IDN; 10 Rehabilitation Medicine, University of Malaya Medical Centre, Kuala Lumpur, MYS; 11 Rehabilitation Medicine, University of Malaya, Kuala Lumpur, MYS; 12 Research, Adventist University of the Philippines, Silang, PHL; 13 Neurosurgery, Bareun Neurosurgery Clinic, Cheongju-si, KOR; 14 Pain Medicine, Miso Pain Clinic, Suwon, KOR; 15 Pain Management, Faculty of Medicine, The Chinese University of Hong Kong, New Territories, HKG; 16 Pain Management, Faculty of Medicine, The University of Hong Kong, Hong Kong, HKG; 17 Musculoskeletal Medicine, The Board of Clinical Research, The Hong Kong Institute of Musculoskeletal Medicine, Kowloon, HKG

**Keywords:** adinizer, cervical myelopathy, disc protrusion, intradiscal injection, mfat, minimally manipulated adipose tissue, regenerative therapy

## Abstract

Cervical myelopathy is a progressive condition caused by spinal cord compression, for which surgical decompression is considered the standard treatment. However, alternative minimally invasive options are needed for patients who decline surgery. We report the case of a 53-year-old female who presented with progressive hand clumsiness, lower extremity weakness, and gait disturbance. She was diagnosed with cervical myelopathy at three tertiary referral centers, where surgical decompression was recommended, but she declined surgery. Imaging revealed a multilevel disc protrusion with spinal cord compression and signal change. An initial fluoroscopy-guided provocative discography with contrast was performed at C4-5, C5-6, and C6-7. No fluoroscopic evidence of extra-annular leakage was observed. Following intradiscal confirmation, lidocaine was administered as part of the diagnostic procedure, which reproduced the patient’s typical symptoms at C4-5 and C5-6 and was followed by temporary symptom relief, thereby identifying the clinically relevant segments. Subsequently, minimally manipulated adipose tissue (MFAT) was injected into the C4-5 and C5-6 discs under fluoroscopic guidance. At six months, follow-up radiographs suggested possible partial restoration of disc space height at the treated levels, although this observation was qualitative and may have been influenced by positional variation. Clinically, the visual analog scale (VAS) improved from 8 to 3, the Neck Disability Index (NDI) improved from 28 to 7, and the modified Japanese Orthopaedic Association (mJOA) score improved from 14 to 18 at one year. Sustained clinical improvement was observed at three-year follow-up. This case suggests that intradiscal MFAT injection may provide sustained clinical improvement, with possible qualitative radiographic interval change, in cervical myelopathy when disc protrusion is the primary contributing factor, and may represent a potential minimally invasive alternative in carefully selected patients who decline surgical intervention.

## Introduction

Cervical myelopathy is a progressive degenerative condition caused by spinal cord compression, most commonly resulting from intervertebral disc protrusion, osteophyte formation, or ligamentous hypertrophy. Patients typically present with hand clumsiness, gait disturbance, and motor weakness, reflecting both upper motor neuron and long tract involvement [1]. Diagnosis is primarily based on clinical findings supported by imaging studies, particularly magnetic resonance imaging (MRI), which can reveal spinal cord compression and intramedullary signal changes.

As a progressive condition, cervical myelopathy requires timely diagnosis and appropriate intervention, and treatment strategies have evolved from a predominantly surgical approach toward more individualized management. Current management ranges from conservative monitoring to surgical decompression, depending on symptom severity and progression. Treatment decisions are guided by careful correlation between clinical findings and imaging at the level of pathology.

Surgical decompression is widely considered the standard treatment, especially in patients with progressive neurological deficits. However, some patients are either unwilling or unsuitable for surgery, highlighting the need for alternative minimally invasive therapeutic options [2].

Recently, regenerative medicine approaches, including stem cell-based therapies, have gained attention for the treatment of degenerative spinal disorders [3-5]. Among these, minimally manipulated adipose tissue (MFAT) has been proposed as a potential therapeutic option due to its anti-inflammatory and regenerative properties.

## Case presentation

A 53-year-old female presented with several months of progressive hand clumsiness and lower extremity weakness without a history of trauma. She had been previously diagnosed with cervical myelopathy at three tertiary referral centers, where surgical decompression was consistently recommended. However, the patient declined surgery and sought alternative treatment options. The patient had no history of trauma, inflammatory arthritis, or systemic rheumatologic disease. There was no known history of occupational exposure to repetitive cervical loading.

Neurological examination revealed preserved higher cortical function. Deep tendon reflexes were hyperactive in both upper and lower extremities, including C5-C7 and L4-S1 levels. Hoffmann’s sign was positive, while Babinski's sign and ankle clonus were absent [6]. Motor weakness was noted in elbow flexion and wrist extension, as well as hip flexion, with Medical Research Council (MRC) grades of IV/IV bilaterally. The patient also demonstrated lower extremity weakness and gait disturbance. There were no abnormalities in bowel or bladder function.

On physical examination, cervical range of motion was limited (extension 30°, rotation 30° bilaterally). Her baseline visual analog scale (VAS) score was 8, the Neck Disability Index (NDI) was 28, and the modified Japanese Orthopaedic Association (mJOA) score was 14, corresponding to moderate myelopathy [7,8].

**Figure 1:**
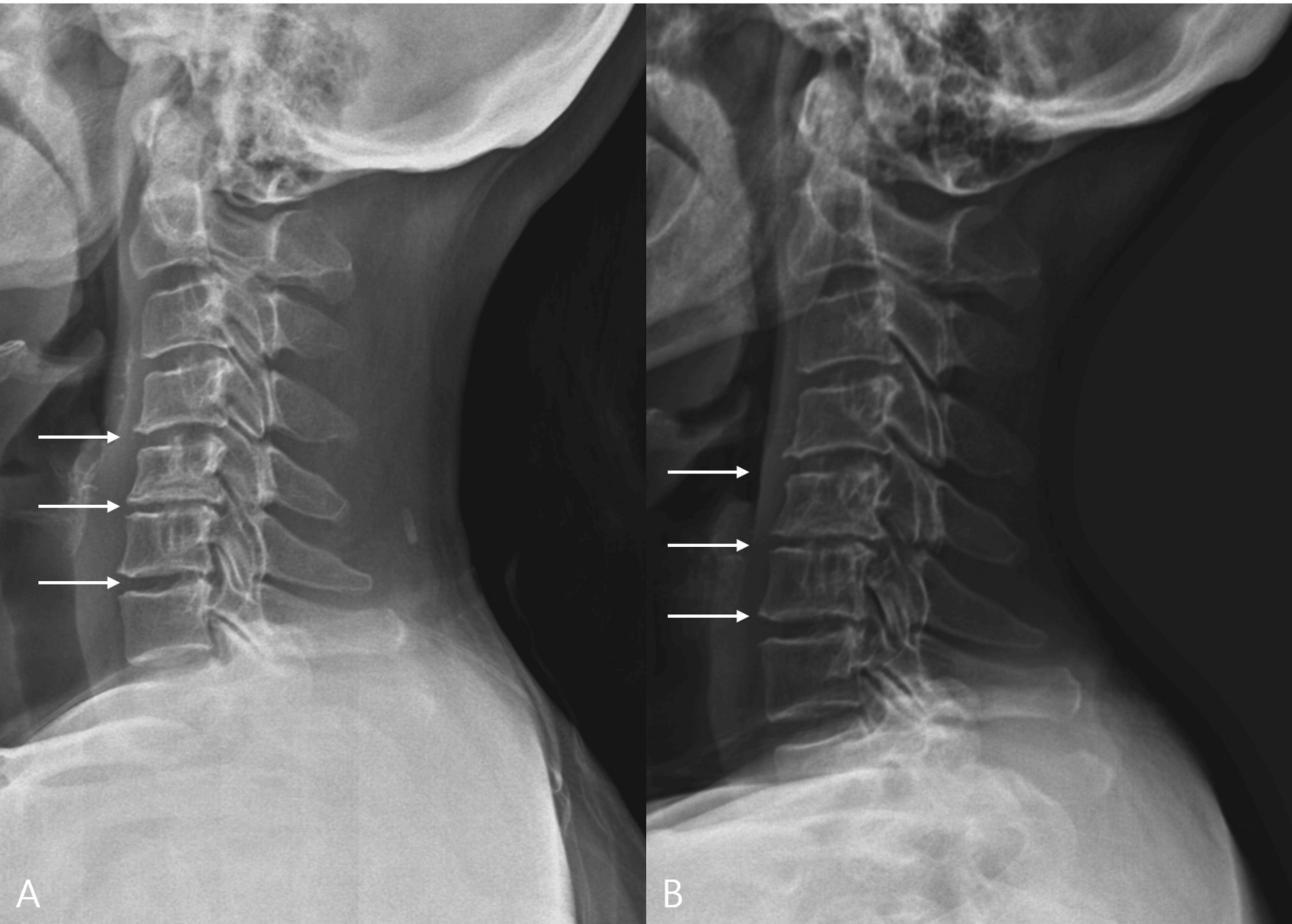
Lateral cervical spine radiographs at baseline and six-month follow-up. (A) Initial radiograph demonstrating multilevel disc space narrowing at C4-5, C5-6, and C6-7 (arrows). (B) Six-month follow-up radiograph of the same levels (arrows), showing relative preservation of disc spaces without clear radiographic progression and suggesting a possible subtle interval increase in disc height at the treated levels; however, this qualitative impression may have been influenced by positional variation.

At baseline, plain radiography demonstrated disc space narrowing at the C4-5 and C5-6 levels (Figure 1A). At the six-month follow-up, follow-up radiographs suggested possible partial restoration of disc space height at the same levels (Figure 1B), although this qualitative finding may have been influenced by positional variation.

**Figure 2:**
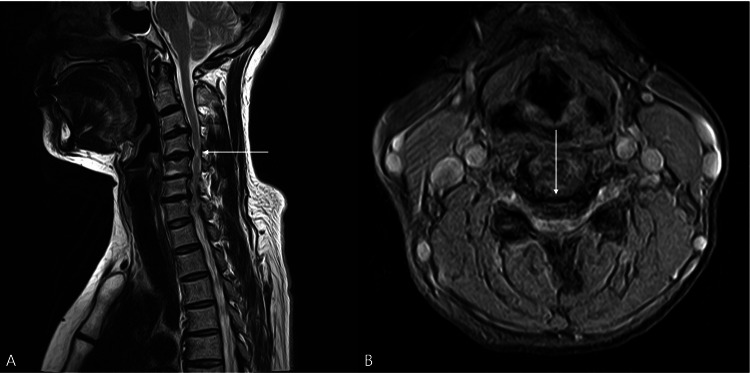
Sagittal and axial T2-weighted magnetic resonance imaging of the cervical spine. (A) Sagittal T2-weighted image demonstrates multilevel disc protrusion from C4 to C7 with associated spinal cord compression. Increased intramedullary signal intensity at the C4 level is observed, consistent with myelopathic change (white arrow). (B) Axial T2-weighted image at the C4 disc level, corresponding to the site of intramedullary signal change, demonstrates focal central canal narrowing with anterior disc protrusion causing direct ventral compression of the spinal cord (white arrow).

MRI demonstrated multilevel disc protrusion from C4 to C7 with associated spinal cord compression and intramedullary signal change at the C4 level (Figure 2).

Although radiographic narrowing was observed across multiple levels (C4-7), the most clinically relevant pathology was identified at the C4-5 level. This level was selected as the primary treatment target based on the correlation between clinical findings and imaging.

**Figure 3:**
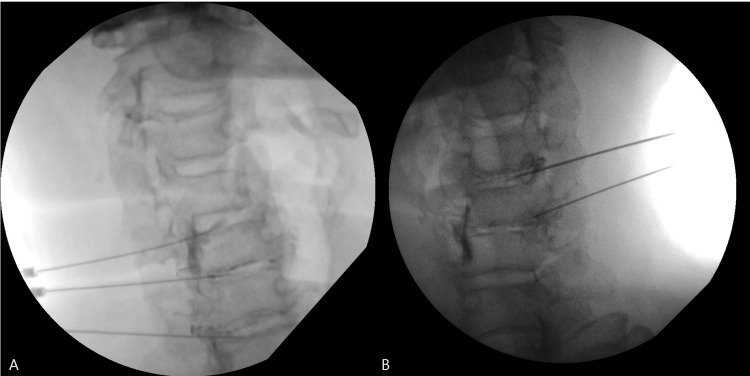
Fluoroscopy-guided intradiscal diagnostic and therapeutic procedures. (A) Initial formal fluoroscopic discography performed at C4-5, C5-6, and C6-7 using contrast medium, followed by intradiscal lidocaine administration after confirmation of intradiscal containment. No fluoroscopic evidence of extravasation was observed. Concordant symptom reproduction was obtained at C4-5 and C5-6, followed by temporary symptom relief. (B) Subsequent intradiscal injection of minimally manipulated adipose tissue (MFAT, 1 mL per level) at C4-5 and C5-6 after symptom recurrence.

Considering that disc pathology was thought to be the primary contributor to canal encroachment, a formal fluoroscopy-guided discographic evaluation was first performed at C4-5, C5-6, and C6-7 using contrast medium. No fluoroscopic evidence of extra-annular leakage was observed during this diagnostic step. After intradiscal containment had been confirmed, lidocaine was administered as part of the same diagnostic procedure. Concordant reproduction of the patient’s typical symptoms was elicited at C4-5 and C5-6, thereby identifying these levels as the primary symptomatic segments. In contrast, no concordant symptom reproduction was observed at C6-7 despite radiographic degeneration (Figure 3A).

The patient experienced temporary symptom relief after this initial diagnostic procedure, further supporting the role of disc pathology at the selected levels. However, symptoms recurred several weeks later. Based on these findings, MFAT, processed using an Adinizer system (Tissue Regeneration Technologies LLC, Weston, FL, USA), was subsequently injected intradiscally at C4-5 and C5-6 (1 mL per level) under fluoroscopic guidance (Figure 3B) [9]. A cervical collar was applied after the procedure, and the patient was instructed in neck muscle energy technique (MET) exercises.

At the six-month follow-up, follow-up radiographs suggested possible partial restoration of disc space height at the treated levels (Figure 1B), although this qualitative impression may have been influenced by positional variation. Clinically, the VAS score improved to 5 at six months and further decreased to 3 at 12 months. The NDI improved from 28 to 7, and the mJOA score improved from 14 to 18 over the same period. At follow-up, deep tendon reflexes normalized, and pathological reflexes, including Hoffmann’s sign, were no longer observed. Motor strength improved to normal (MRC grade V), consistent with clinical and functional recovery. Long-term follow-up at three years revealed sustained clinical improvement without progression of neurological symptoms. As the patient resided at a distant location, follow-up was conducted via telephone interview.

**Figure 4:**
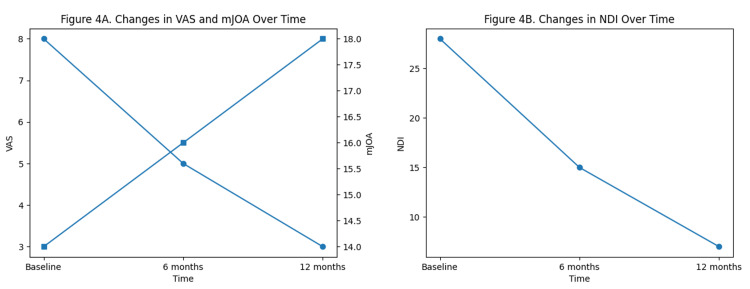
Changes in clinical outcomes over time. (A) Dual-axis line graph demonstrating progressive improvement in visual analog scale (VAS, left axis) and modified Japanese Orthopaedic Association (mJOA, right axis) scores over a one-year follow-up period. (B) Line graph showing improvement in Neck Disability Index (NDI) scores over the same period.

Clinical outcomes demonstrated progressive improvement over time, as illustrated in Figure 4.

## Discussion

This case describes observed clinical improvement after intradiscal MFAT injection in a highly selected patient with cervical myelopathy associated with disc pathology who declined recommended surgical decompression.

Stem cell-based therapies have been increasingly explored in the treatment of degenerative spinal disorders [3,4,10]. However, the majority of existing studies have focused on lumbar disc disease, with relatively limited research addressing cervical disc pathology [11,12]. Furthermore, among regenerative approaches, studies specifically investigating MFAT in the cervical spine are very limited.

To our knowledge, while preclinical studies, including porcine models, have demonstrated the regenerative potential of adipose-derived therapies in intervertebral discs, there are currently no clinical reports describing the application of MFAT in cervical myelopathy [13]. In this context, the present case may provide preliminary clinical insight into its utility.

Cervical myelopathy is a multifactorial condition, with spinal cord compression often arising from a combination of disc protrusion, osteophytes, and ligamentous hypertrophy. In the present case, the clinical and imaging findings suggested that disc pathology was a major contributor to ventral canal encroachment, and treatment was directed accordingly. Importantly, target-level selection was not based on imaging alone. Formal fluoroscopy-guided discographic evaluation with contrast was performed prior to treatment, and no fluoroscopic evidence of extra-annular leakage was observed. After confirmation of intradiscal containment, lidocaine was administered as part of the diagnostic procedure, and concordant symptom reproduction was obtained at C4-5 and C5-6 but not at C6-7. These findings strengthened the identification of the clinically relevant levels, although they should not be interpreted as establishing broader procedural safety or therapeutic efficacy.

In particular, this case raises the possibility that, in carefully selected patients in whom disc protrusion appears to be a major contributor to canal compromise, intradiscal biologic intervention may warrant further cautious study. However, no conclusion regarding safety, efficacy, or general clinical applicability can be drawn from a single case.

The follow-up radiographs at six months suggested a possible qualitative interval change in disc space height, and sustained clinical improvement was observed over a three-year follow-up period, including improvement in mJOA score. Together, these findings suggest temporal coexistence of radiographic and clinical change after MFAT injection, although no causal inference can be drawn from a single case. The observed improvement in mJOA score, motor findings, and pathological reflexes is consistent with neurological improvement over time, but the relative contributions of the intradiscal procedure, the natural course, adjunctive care, and other unmeasured factors cannot be determined. The underlying mechanism, therefore, remains speculative.

Nevertheless, this study has several important limitations. First, although formal fluoroscopy-guided discography with contrast was performed prior to treatment, and no fluoroscopic evidence of extra-annular leakage was observed, CT discography was not performed. Therefore, annular integrity and possible occult extra-annular contrast spread could not be fully characterized beyond fluoroscopic assessment. This is an important limitation when interpreting procedural safety, particularly in a patient with pre-existing cervical cord compression. Second, non-contrast CT imaging was not obtained, which limits assessment of bony contributors such as osteophytes or ossification. Third, a follow-up MRI was not available, limiting objective evaluation of interval changes in disc morphology, canal dimensions, and spinal cord signal alteration. As a result, the interpretation of structural change relies primarily on plain radiographic findings and clinical outcomes. In addition, this was a single case report, so the findings are not generalizable and may not be reproducible in a broader population. Finally, interval changes in disc space height were assessed qualitatively rather than with standardized millimeter-based measurements, which limits radiographic precision.

A further point of caution is that, in the setting of cervical myelopathy, any intradiscal procedure must be interpreted in light of the theoretical risk of extra-annular spread. If local anesthetic or biologic material were to extend beyond a disrupted annulus, neurologic risk could theoretically be amplified in the presence of pre-existing cord compression. Although no fluoroscopic extravasation was observed in this case, this single observation should not be interpreted as establishing procedural safety for routine use in similar patients.

Despite these limitations, this case may be of hypothesis-generating value by illustrating the importance of careful clinicoradiologic correlation and level-specific diagnostic assessment in a patient with presumed disc-driven cervical myelopathy who declined surgery. However, it should not be interpreted as evidence supporting routine intradiscal biologic treatment in cervical myelopathy.

## Conclusions

In this case, improvement in pain and neurological function, as reflected by VAS and mJOA scores, was observed following intradiscal MFAT injection. Follow-up radiographs also suggested a possible interval change in disc space height, although this finding should be interpreted cautiously because it was not based on standardized quantitative measurement.
